# Impact of Continuous Veno-Venous Hemodiafiltration on Thyroid Homeostasis in Critically Ill Patients

**DOI:** 10.3390/jcm14155542

**Published:** 2025-08-06

**Authors:** Alicja Filipczyk, Magdalena A. Wujtewicz, Michał Okrągły, Karol P. Steckiewicz

**Affiliations:** Department of Anesthesiology and Intensive Therapy, Faculty of Medicine, Medical University of Gdańsk, 80-211 Gdańsk, Poland

**Keywords:** thyroxine, deiodinase activity, hypothyroidism, CRRT, non-thyroidal illness syndrome

## Abstract

**Background**: Patients in Intensive Care Units (ICUs) often develop non-thyroidal illness syndrome. Potentially, thyroid hormones may be removed during continuous veno-venous hemodiafiltration (CVVHDF), as their molecular size is smaller than the filter pores’ cutoff. The study’s main aim was to assess whether the serum concentration of thyroid hormones changes over time during CVVHDF. **Methods**: This was a prospective observational trial that included 30 patients treated in an ICU. All patients developed acute kidney injury (AKI) and had clinical indications for implementation of CVVHDF. Blood samples were collected before initiation of CVVHDF and at 1, 2, 3, 6, 9 and 12 days after. The last sample was collected three days after CVVHDF withdrawal. Thyroid function was evaluated by determining the serum concentration of TSH, thyrotropin-releasing hormone (TRH), free triiodothyronine (fT_3_), free thyroxine (fT_4_), total triiodothyronine (tT_3_), total thyroxine (tT_4_) and reverse triiodothyronine (rT_3_). We additionally calculated the total activity of peripheral deiodinases (G_D_) using a mathematical model. **Results**: TRH and TSH levels remained mostly within normal ranges. fT4 and tT4 were in normal range or slightly below. In contrast, fT3 and tT3 were undetectably low in most patients throughout. Reverse T3 levels remained within normal limits. There were no statistically significant changes in any thyroid hormone levels over the CVVHDF treatment period. The calculated peripheral G_D_ activity was lower than normal, but importantly, it did not change significantly over time. **Conclusions**: Thyroid hormones are not lost due to hemodiafiltration. Decreased deiodinases activity is responsible for alterations in serum concentrations of thyroid hormones in patients during CVVHDF.

## 1. Introduction

In life-threatening conditions, significant changes in homeostasis take place. These include endocrine adaptations in the hypothalamic–anterior pituitary–peripheral hormone axes [1]. Thus, thyroid dysfunction is present in most patients in the intensive care unit (ICU) [2]. This phenomenon is often referred to as non-thyroidal illness syndrome (NTIS). Decreased levels of triiodothyronine (T3) with normal or mildly decreased thyroid-stimulating hormone (TSH) are observed in NTIS [3]. It has been shown that serum T_3_ can decrease in as little as two hours after the onset of acute stress [4]. The scale of this change correlates with the severity of the disease and may serve as a negative prognostic factor [5,6,7]. The pathophysiology of NTIS is not fully understood; however, factors such as downregulation of the hypothalamic–pituitary–thyroid axis [8], changes in the expression of proteins regulating thyroid hormone metabolism [9], changes in deiodinase activity [10], decreased caloric intake [11] and drug side effects [12] are being studied. Some researchers hypothesized that NTIS may be an organism’s way of conserving energy needed for the immune response and healing process [8]. The absence of clear guidelines for NTIS management in critically ill patients highlights a significant clinical dilemma [3].

Also, acute kidney injury (AKI) is a major clinical problem in the ICU cohort. According to RIFLE criteria, AKI is diagnosed based on serum creatinine levels and urine output [13] and may be present in up to a quarter of ICU patients, with mortality as high as 60% [14]. Septic shock, hypovolemia, hypotension, rhabdomyolysis and surgery are among the main causes of AKI in critically ill patients [15]. Many individuals who develop AKI will require renal replacement therapy (RRT). Studies report that even up to 25% of patients hospitalized in ICUs required RRT [16,17]. In the ICU setting, continuous veno-venous hemodiafiltration (CVVHDF) is the preferred method of RRT. It consists of hemodialysis (HD) based on diffusion as well as hemofiltration (HF) based on convection [18]. During HF, large molecules such as proteins and cytokines are carried across the membrane with the solvent. In contrast, during HD, small molecules like ions, creatinine and urea are removed until equilibrium on each side of the membrane is reached [18]. During CVVHDF, molecules up to 10 kilodaltons may be removed. Although CVVHDF filters have a theoretical pore cutoff of ~30–40 kDa, effective solute clearance is typically limited to molecules < 10 kDa. Both T_3_ (~650 Da) and T_4_ (~750 Da) molecular sizes are much smaller than the filter cutoff; thus, hypothetically, they may be lost to the dialysate. Importantly, NITS and AKI may overlap in a significant amount of critically ill patients, and a decrease in T3 correlates with rising creatinine, suggesting that thyroid function declines as renal failure worsens [19].

The aim of this study was to determine whether the serum concentrations of thyroid hormones and related parameters change over time during CVVHDF and after its discontinuation, given that thyroid hormone molecules are smaller than the filter pores and could theoretically be lost through the filter. Hitherto, no data have addressed this specific issue. The evidence generated may improve our understanding of thyroid homeostasis in critically ill patients undergoing CVVHDF.

## 2. Materials and Methods

This observational, prospective, single-center trial was conducted in Gdańsk, Poland, between February 2019 and February 2020, at the Department of Anesthesiology and Intensive Therapy, the Medical University of Gdańsk. The protocol was approved by the Independent Bioethics Committee for Scientific Research at the Medical University of Gdańsk (approval numbers NKBBN/551/2017-2018 and NKBBN/551-217/2021), and the trial adhered to Good Clinical Practice (GCP) guidelines. It was registered on ClinicalTrials.gov (NCT04767763). The full protocol is available upon request from the corresponding author.

### 2.1. Participants

Thirty patients treated in the University Clinical Center in Gdansk ICU were enrolled in the study. All patients had acute kidney injury (AKI) necessitating CVVHDF. All patients were treated with CVVHDF with citrate anticoagulation on a Baxter Prismaflex system (Deerfield, Chicago, IL, USA) with ST-150 filter (Deerfield, Chicago, IL, USA), dialysate, and replacement fluid compositions based on the patient’s clinical status. All patients underwent high-volume CVVHDF with a dose > 30 mL kg^−1^ h^−1^. Clinical data recorded included demographic information and illness severity scores (Simplified Acute Physiology Score II [SAPS II]; Acute Physiology and Chronic Health Evaluation II, [APACHE II]; and the associated predicted death rate, [PDR]). Due to the observational character of the study, the institutional review board waived the need for patients’ informed consent. Inclusion criteria were (i) age > 18 years old, (ii) treatment in ICU and (iii) clinical indication for CVVHDF. Exclusion criteria were (i) preexisting end-stage chronic kidney disease and (ii) preexisting hypothyroidism. Patients taking drugs that may impact thyroid homeostasis or interfere with thyroid-related parameter measurements were also excluded from the study.

### 2.2. Study Protocol

Blood samples were collected at baseline (immediately before CVVHDF initiation), then on days 1, 2, 3, 6, 9 and 12 of therapy. If CVVHDF was stopped earlier, sampling stopped accordingly. The final sample was obtained 3 days after CVVHDF discontinuation for those who survived or recovered renal function (denoted as ‘endpoint’). At designated time points, blood samples were collected in 5 mL tubes without the addition of an anticoagulant or preservative. For evaluating thyroid function, serum concentrations of (i) thyroid-stimulating hormone (TSH), (ii) thyrotropin-releasing hormone (TRH), free triiodothyronine (fT_3_), free thyroxine (fT_4_), total triiodothyronine (tT_3_), total thyroxine (tT_4_) and reverse triiodothyronine (rT_3_) were determined. After blood collection, TSH, fT3 and fT4 concentrations were determined without delay. For TRH, tT_3_, tT_4_ and rT_3,_ serum was collected and frozen (−80 °C), and measurements were done at the end of the study. All analyses were performed in the Central Clinical Laboratory of the University Clinical Center in Gdansk. Commercially available ELISA tests were used to determine levels of rT3 (Tecan, IBL International GmbH, Hamburg, Germany) and TRH (Cusabio, Houston, TX, USA). Commercially available chemiluminescent microparticle immunoassays (CMIA) were used to determine levels of TSH (Alinity i TSH, Abbott, Ireland, IL, USA), fT3 (Alinity i Free T3, Abbott, Ireland), fT4 (Alinity i Free T4, Abbott, Ireland, IL, USA), tT3 (Architect T3, Abbott, Ireland, IL, USA) and tT4 (Architect T4, Abbott, Ireland, IL, USA). All analytical tests were performed according to manufacturers’ guidelines. GD was calculated using the method of Dietrich et al., which incorporates equilibrium fT4 and tT3 levels along with kinetic constants to estimate overall deiodinase activity. The formula accounts for T3 clearance rate (*β*_31_), deiodinase type 1 Michaelis constant (*K_M_*_1_) and a dilution factor for T3 distribution (*α*_31_) [20]:

$$G_{D}=\frac{\beta_{31}\left(K_{M1}+\left[fT_{4}\right]\right)\left[tT_{3}\right]}{\alpha_{31}\left[fT_{4}\right]}$$

*β*_31_—clearance exponent for T3 (8 × 10^−6^ s^−1^);
*K_M_*_1_—dissociation constant of type-1-deiodinase (5 × 10^−7^ mol/L);
*α*_31_—dilution factor for T3 (0.026 L^−1^);
*G_D_*—normal reference range is 20–60 nmoL s^−1^.

### 2.3. Statistical Analyses

The interim analyses for futility or efficacy and sub-group analysis were not included in the study protocol. The primary endpoint was the change in thyroid hormone levels over time during CVVHDF. Statistical analysis was performed using Statistica 13.0 PL Software (Dell Statistica, Tulsa, OK, USA). If the value fell below the lower limit of quantification (LOQ) for statistical analysis, it was replaced with LOQ/2, following a widely acknowledged protocol [21]. The normality of the continuous data distribution was tested with the Shapiro–Wilk W test. The Friedman test with Dunn’s post hoc analysis was used to compare nonparametric data. A *p*-value < 0.05 was considered statistically significant.

## 3. Results

The study included 30 patients, 22 male (73%) and 8 female (27%), with a mean age of 67.1 ± 11.7 years. Detailed study population characteristics are presented in Table 1.

As shown in Table 1, patients were severely ill on admission (mean APACHE II score 27 ± 7.6; predicted mortality ~55%) and CVVHDF was initiated early (median 1 day [IQR 1–3] from ICU admission) for an average duration of ~8.5 days.

**Figure 1:**
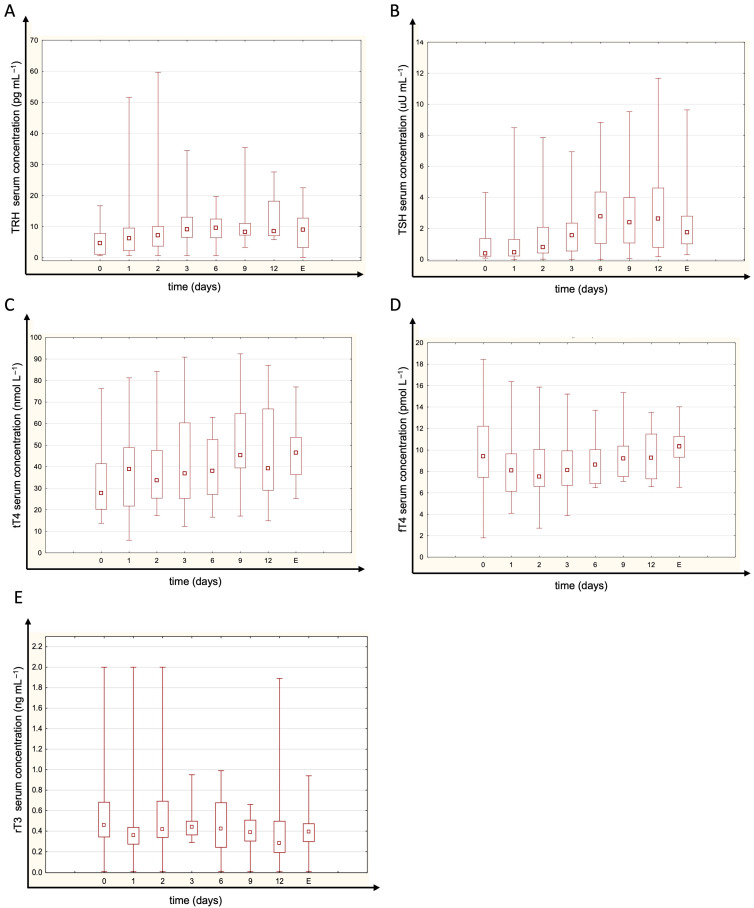
The serum concentrations of (**A**) thyrotropin-releasing hormone (TRH), (**B**) thyroid-stimulating hormone (TSH), (**C**) total thyroxine (tT_4_), (**D**) free thyroxine (fT_4_) and (**E**) reverse triiodothyronine (rT_3_) at given time points (E—sample obtained 3 days after CVVHDF termination). Free T3 and total T3 are not plotted due to most values being below the detection limit. There were no significant differences between time points (Friedman test *p* = 0.156 for TRH; *p* = 0.061 for TSH; *p* = 0.399 for tT_4_; *p*= 0.291 for fT_4_, *p* = 0.747 for tT_4_). Normal ranges are <40 pg mL^−1^ for TRH, 0.35–4.94 uM mL^−1^ for TSH, 62.68–150.83 mmol L^−1^ for tT_4_, 9.01–19.05 pmol L^−1^ for fT_4_ and 0.072–0.309 ng mL^−1^ for rT_3_. Box span values represent the 25–75 percentile range, whiskers show the minimum–maximum range, and the square in the box represents the median. N_0_ = 30; N_1_ = 30; N_2_ = 26; N_3_ = 25; N_6_ = 18; N_9_ = 15; N_12_ = 10; N_E_ = 12.

**Figure 2:**
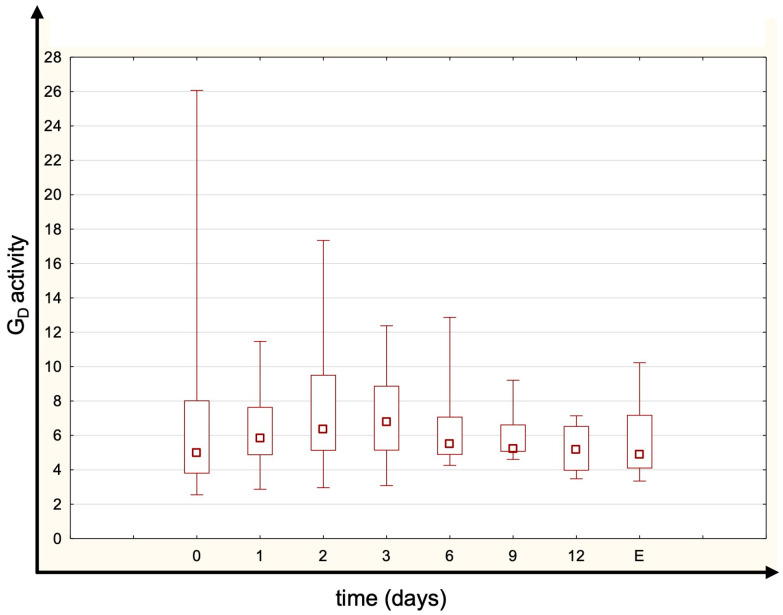
The summed activity of peripheral deiodinases (G_D_) at different time points (E—sample obtained 3 days after CVVHDF termination). There were no significant differences between time points (Friedman test, *p* = 0.259). Box span values represent the 25–75 percentile range, whiskers show the minimum–maximum range, and the square in the box represents the median. N_0_ = 30; N_1_ = 30; N_2_ = 26; N_3_ = 25; N_6_ = 18; N_9_ = 15; N_12_ = 10; N_E_ = 12.

Most patients had TRH and TSH levels within the normal range. The concentrations of fT4 and tT4 were generally normal or just below normal limits (Figure 1). fT3 and tT3 were unmeasurably low in the majority of patients at all time points (below assay detection limits of 1.46 pmol/L for fT3 and 0.305 ng/mL for tT3). Most patients’ rT3 concentrations remained within the normal range (Figure 1). There were no statistically significant differences in thyroid hormone levels between measured time points. No significant temporal variation in the rT3/tT3 ratio was observed, suggesting stable thyroid hormone metabolism during CVVHDF. The estimated global deiodinase activity (G_D_) was below the normal range (indicating reduced peripheral conversion), but it remained essentially constant throughout CVVHDF (no significant differences between time points, *p* = 0.259; Figure 2). The median (IQR) GD activity was 4.86 (3.97–6.57) at baseline, increased to 6.02 (4.97–7.64) on day 1, 6.26 (5.52–7.46) on day 2, and peaked at 6.66 (5.56–8.13) on day 3 before declining to 5.54 (5.25–7.09) on day 6. However, these changes over time did not reach statistical significance. The normal range for G_D_ activity is 20–60 nmol s^−1^ [20].

## 4. Discussion

To our knowledge, no previous data exist on whether thyroid hormones can be removed during CVVHDF. Our findings confirm that critically ill ICU patients with AKI exhibit the characteristic NTIS pattern of thyroid hormone alterations, and importantly, CVVHDF did not further impact or alter these hormone levels.

Our cohort had typical disturbances characteristic of NTIS [3]. We reported that fT_4_ levels were close to normal values, whereas fT_3_ levels were significantly decreased, consistent with observations in other critically ill populations [22]. Furthermore, Rothberger et al. [23] found that low fT3 in ICU patients was not accompanied by elevated TSH, which aligns with our findings (TSH remained in normal range despite low fT3). Since the majority of T3 (~80%) is produced by peripheral conversion of T4, changes in deiodinase activity can cause more T4 to be converted into inactive rT3 rather than active T3 [24]. In NTIS, D1 and D2 deiodinases activity to convert T4 into T3 decreases. The decrease in D2 deiodinase activity depends on the tissue and severity of the illness. Concomitantly, D3 deiodinase, which converts T_4_ into metabolically inactive rT_3_, is overexpressed. However, some of these findings come from animal models, so their translation to human critical illness is uncertain [24,25,26,27]. Our findings of low GD activity, depressed T3 levels and rT3 concentrations within the normal range are consistent with the endocrine profile of NTIS in critical care. In this state, reduced D1 and D2 activity impairs the conversion of T4 into biologically active T3, while D3—which converts T4 into inactive rT3—may be upregulated. This enzymatic shift leads to a reduction in serum T3, with rT3 levels either preserved or mildly increased. While our patients’ rT3 remained within reference limits, this pattern has been documented in acute NTIS and may reflect early or less severe axis suppression. While a simple T_3_ to T_4_ ratio is sometimes used as a surrogate for deiodinase activity, we decided to use a more advanced mathematical model that takes into consideration equilibrium concentrations of fT_4_, fT_3_ and pharmacokinetic constants to determine the sum activity of peripheral deiodinases [20]. The model’s accuracy was previously established in in vivo studies; thus, even though we did not directly measure deiodinase activity, we are confident that the obtained results provide clinically significant data [20]. NTIS in ICU patients has been associated with higher mortality. For instance, Wang et al. have shown that fT3 has the highest prognostic power among thyroid-related parameters to predict ICU mortality. Non-survivors had significantly decreased levels of that hormone. Authors even suggested the implementation of fT_3_ into the APACHE II score to improve its sensitivity towards death prediction [28]. Similar findings were reported by Gutch et al. [29]. Another study by Plikat et al. found that lower fT_4_ may also suggest higher mortality [30]. Whether treating NTIS with thyroid hormone supplementation is beneficial for critically ill patients remains unclear. Brent and Hershman have shown that IV thyroxine supplementation in NTIS was not beneficial; it raised fT4 levels while further suppressing TSH [31]. In contrast, other studies in post-cardiac surgery patients have shown that thyroid hormone supplementation can reduce complications and even mortality [32,33,34].

Continuous RRT can remove not only small solutes (ions, urea, creatinine) but also various other substances, including drugs, vitamins and certain biomarkers [35,36]. Lim et al. [37] reported a case of thyroid storm in which thyroid hormone levels decreased over consecutive days of continuous renal replacement therapy (CRRT). However, that case fundamentally differs from our study in several key aspects. First, the patient had severe thyrotoxicosis with massive excess of free and total thyroid hormones, in contrast to our cohort, which exhibited NTIS-related hormone deficiency. Second, antithyroid medications (e.g., propylthiouracil) were co-administered, directly suppressing hormone synthesis and potentially altering peripheral metabolism. Third, in thyroid storm, the large pool of circulating unbound hormone may overwhelm protein-binding capacity, increasing the free hormone fraction available for clearance. In contrast, NTIS is associated with low or normal levels of free hormones and intact or reduced protein binding, further limiting dialytic removal. Therefore, the findings from Lim et al. should not be extrapolated to critically ill patients with NTIS undergoing CVVHDF [37]. Over 40 years ago, Forest et al. [38] found no significant differences in T_3_, T_4_, fT_4_ or TSH levels measured before vs. after a hemodialysis session in patients with chronic renal failure. However, those patients’ baseline thyroid hormone levels were significantly lower than those in healthy controls [38]. Another relatively old study showed no differences in thyroid hormones in patients undergoing hemodialysis and continuous ambulatory peritoneal dialysis [39]. Nonetheless, the impact of continuous renal replacement therapy on hormone levels is poorly investigated. Bashan et al. [40] have shown that cortisol levels decrease during CRRT. Boer et al. [41] reported that levels of intact (iPTH) and oxidized parathormone (oxPTH) decreased after continuous veno-venous hemofiltration (CVVHF). They did not observe any changes in non-oxidized parathormone levels (noxPTH). However, these hormonal differences could be explained by alterations in calcium balance and reduced oxidative stress during CVVHF, rather than direct hormone loss in the filtrate.

In summary, the decreases in thyroid hormones seen in our patients were not attributable to CVVHDF removing them. This is biologically plausible because thyroid hormones are mostly protein bound, the free hormone fraction available for dialysis is minimal and homeostatic mechanisms may replenish it quickly. Thus, even though T_3_ and T_4_ are small molecules, their effective removal by CVVHDF would be limited. However, our study has some limitations. We did not measure the thyroid hormone-binding proteins that carry ~99% of T4 and T3 in circulation, namely thyroxine-binding globulin (TBG), transthyretin (thyroxine-binding prealbumin, TBPA) and albumin. TBG, a 54 kDa glycoprotein synthesized in the liver, is the principal high-affinity transport protein and binds around 75% of circulating thyroxine and triiodothyronine [42]. The remainder is bound to transthyretin and albumin, both of which have lower binding affinities [42]. Due to this extensive protein binding, molecular weight alone is insufficient to predict removal during CVVHDF. Although T_4_ and T_3_ have small molecular weights (~750 and ~650 Da, respectively), only the free hormone fraction can be cleared across dialysis membranes [43]. Protein-bound hormones are not filtered and, without dissociation from their carriers, clearance remains negligible. This explains why standard dialysis has been shown to remove very little thyroid hormone, despite its low molecular weight [38]. Variations in TBG or other carrier protein levels could influence total thyroid hormone levels and affect the potential for dialytic loss. We did not measure deiodinases activity but determined that variable using a mathematical formula. While this approach is validated, it provides an indirect assessment. Additionally, this was a single-center study with a relatively small sample size (30 patients). However, our cohort size is comparable to those in similar exploratory studies in critical illness [40,41,44,45]. Also, it must be noted that patients were heterogeneous regarding the underlying cause of the AKI, which may impact the results.

## 5. Conclusions

Our findings indicate that CVVHDF does not cause any significant loss of thyroid hormones in critically ill AKI patients. The observed low T_3_ and altered thyroid hormone levels appear to result from decreased peripheral deiodinase activity (due to NTIS) rather than the renal replacement therapy. It remains unclear whether this deiodinase impairment is solely due to the critical illness itself or if other factors also contribute to the thyroid hormone changes.

## Figures and Tables

**Table 1:** Patient characteristics and outcomes. Values are number [%] or mean (SD) and median [IQR].

	Study Group (*N* = 30)
Female sex	N = 8 [27%]
Age, year	67.1 (11.7)
SAPS II score	53 (16.9)
APACHE II score	27.1 (7.6)
PDR	0.59 (0.2)
Days from ICU admission to CVVHDF implementation	1 [1–3]
Length of CVVHDF duration	8.5 [4–13.5]
Reason for ICU admission	Septic shock N = 11 [37%]
	Acute respiratory failure N = 4 [13%]
	Trauma N = 3 [10%]
	Post-resuscitation care N = 3 [10%]
	Acute cardiac failure N = 2 [7%]
	Hematological malignancy N = 2 [7%]
	Hemorrhagic shock N = 1 [3%]
	Acute pancreatitis N = 1 [3%]
	Toxic epidermal necrolysis N = 1 [3%]
	Multiorgan failure N = 1 [3%]
	Pulmonary thromboembolism N = 1 [3%]
Acute Kidney Injury cause	Septic shock N = 21 [70%]
	Exacerbation of chronic kidney disease N = 3 [10%]
	Multiorgan failure N = 2 [7%]
	Rhabdomyolysis N = 2 [7%]
	Hemorrhagic shock N = 1 [3%]
	Unknown N = 1 [3%]
Reason of CVVHDF discontinuation	Recovery of renal function N = 16 [53%]
	Death N = 12 [40%]
	Withdrawal of care due to futility N = 2 [7%]

SAPS II: Simplified Acute Physiology Score II; APACHE II: Acute Physiology and Chronic Health Evaluation II; PDR: predicted death rate; CVVHDF: continuous veno-venous hemodiafiltration; ICU: intensive care unit.

## Data Availability

The datasets are available from the corresponding author on reasonable request.
